# DirectASRM: uncovering allele-specific post-transcriptional RNA modifications through direct RNA sequencing

**DOI:** 10.1093/bioinformatics/btag432

**Published:** 2026-06-22

**Authors:** Jiayin Dai, Yuxin Zhang, Jiayi Li, Jiongming Ma, Kunqi Chen, Jia Meng, Daniel J Rigden, Zhen Wei, Shaofeng Lin, Qingru Xu

**Affiliations:** Department of Biological Sciences and Bioinformatics, Xi’an Jiaotong-Liverpool University, Suzhou, Jiangsu 215123, China; Key Laboratory of Ministry of Education for Gastrointestinal Cancer, School of Basic Medical Sciences, Fujian Medical University, Fuzhou, Fujian 350004, China; Institute of Systems, Molecular and Integrative Biology, University of Liverpool, Liverpool, L7 8TX, United Kingdom; Department of Biological Sciences and Bioinformatics, Xi’an Jiaotong-Liverpool University, Suzhou, Jiangsu 215123, China; Key Laboratory of Ministry of Education for Gastrointestinal Cancer, School of Basic Medical Sciences, Fujian Medical University, Fuzhou, Fujian 350004, China; Institute of Systems, Molecular and Integrative Biology, University of Liverpool, Liverpool, L7 8TX, United Kingdom; Department of Biological Sciences and Bioinformatics, Xi’an Jiaotong-Liverpool University, Suzhou, Jiangsu 215123, China; Key Laboratory of Ministry of Education for Gastrointestinal Cancer, School of Basic Medical Sciences, Fujian Medical University, Fuzhou, Fujian 350004, China; Institute of Systems, Molecular and Integrative Biology, University of Liverpool, Liverpool, L7 8TX, United Kingdom; Department of Biological Sciences and Bioinformatics, Xi’an Jiaotong-Liverpool University, Suzhou, Jiangsu 215123, China; Key Laboratory of Ministry of Education for Gastrointestinal Cancer, School of Basic Medical Sciences, Fujian Medical University, Fuzhou, Fujian 350004, China; Institute of Systems, Molecular and Integrative Biology, University of Liverpool, Liverpool, L7 8TX, United Kingdom; Key Laboratory of Ministry of Education for Gastrointestinal Cancer, School of Basic Medical Sciences, Fujian Medical University, Fuzhou, Fujian 350004, China; Fujian Key Laboratory of Tumor Microbiology, Department of Medical Microbiology, School of Basic Medical Sciences, Fujian Medical University, Fuzhou, Fujian 350004, China; Department of Biological Sciences and Bioinformatics, Xi’an Jiaotong-Liverpool University, Suzhou, Jiangsu 215123, China; Institute of Systems, Molecular and Integrative Biology, University of Liverpool, Liverpool, L7 8TX, United Kingdom; AI University Research Centre, Xi’an Jiaotong-Liverpool University, Suzhou, Jiangsu 215123, China; Institute of Systems, Molecular and Integrative Biology, University of Liverpool, Liverpool, L7 8TX, United Kingdom; Department of Biological Sciences and Bioinformatics, Xi’an Jiaotong-Liverpool University, Suzhou, Jiangsu 215123, China; Institute of Life Course and Medical Sciences, Xi’an Jiaotong-Liverpool University, Suzhou, Jiangsu 215123, China; Key Laboratory of Ministry of Education for Gastrointestinal Cancer, School of Basic Medical Sciences, Fujian Medical University, Fuzhou, Fujian 350004, China; Department of Biological Sciences and Bioinformatics, Xi’an Jiaotong-Liverpool University, Suzhou, Jiangsu 215123, China; Bioinformatics Interdepartmental Program, University of California, Los Angeles, Los Angeles, CA 90095, United States

## Abstract

**Summary:**

We developed DirectASRM, a comprehensive database for the systematic identification, integration, and annotation of allele-specific RNA modifications (ASRMs) from direct RNA sequencing data. DirectASRM enables single-base, transcript-level detection of ASRMs across multiple RNA modification types, diverse organisms and condition-specific contexts. The database further evaluates the confidence of each ASRM–SNP pair association within isoform context by jointly considering statistical evidence of allelic modification imbalance and independent support from external next-generation sequencing (NGS) – based RNA modification resources. DirectASRM also provides extensive functional annotations for ASRMs and their associated variants, including intra-sample transcript-level allele-specific expression (ASE) and allele-specific splicing, as well as additional post-transcriptional regulatory features such as miRNA binding, circRNA, RNA–protein interactions, and disease relevance. Overall, DirectASRM serves as a comprehensive resource that supports systematic investigation of the potential functional impact of genetic variants in epitranscriptomic regulation.

**Availability and implementation:**

DirectASRM database is freely accessible at http://modinfor.com/DirectASRM/. DirectASRM pipeline is available at GitHub (https://github.com/jiayin1101/DirectASRM_pipeline) and Zenodo (DOI: https://doi.org/10.5281/zenodo.19876077).

## 1 Introduction

High-throughput sequencing has enabled the identification of vast numbers of single-nucleotide polymorphisms (SNPs) (Gonzaga-Jauregui *et al.* 2012); however, distinguishing variants with functional consequences remains challenging. Compared to traditional association studies, evaluating allelic imbalance at heterozygous loci enables the detection of functional variant effects with improved sensitivity, finer resolution, and lower sample size requirements (Buyan *et al.* 2025). Starting from early studies on allele-specific gene expression (ASE) (Yan *et al.* 2002, Ge *et al.* 2009), the analysis of allele-specific variants has become a major area of study at the intersection of genetics and functional genomics. Over the past decade, numerous strategies have been developed to detect allelic imbalance across various omics layers, such as allele-specific binding (ASB) of transcription factor or RNA-binding proteins, allele-specific DNA methylation (ASM), and allele-specific RNA secondary structure (ASRS) (Bahrami-Samani and Xing 2019, Abramov *et al.* 2021, Glinos *et al.* 2022, Zhou *et al.* 2022, Xu *et al.* 2024). Together, these studies demonstrate the power of allele-specific frameworks to uncover cis-regulatory effects that are often obscured in bulk analyses.

The epitranscriptome represents an additional and increasingly recognized regulatory layer of gene expression (He 2010, Saletore *et al.* 2012), with more than 170 post-transcriptional RNA modifications (RMs) implicated in diverse biological processes and disease mechanisms (Esteve-Puig *et al.* 2020, Jones *et al.* 2020, Zhang *et al.* 2020). To investigate the impact of genetic variation on RNA modifications, several resources have applied in-silico mutations, next-generation sequencing (NGS)-based strategies, and more recently AI-driven approaches to study RM-associated variants (Chen *et al.* 2021, Luo *et al.* 2021, Song *et al.* 2023, Huang *et al.* 2024, 2025); however, these approaches are either prediction-based or constrained by isoform ambiguity inherent to short-read sequencing data. More recently, RMpore characterized haplotype-biased RNA modification sites in human and mouse using haplotype-based phasing of long-read sequencing data (Lin *et al*. 2026). While haplotype phasing enables parental allele assignment, it is not strictly required for detecting local allelic imbalance in RNA modification. Importantly, existing resources do not systematically evaluate the association between individual genetic variants and allele-specific RNA modification events.

To address these limitations, we developed DirectASRM, a comprehensive database for the systematic identification, integration, and annotation of allele-specific RNA modifications from direct RNA sequencing data. DirectASRM enables single-base, transcript-level detection of ASRMs across multiple RNA modification types, species, and condition-specific contexts. Crucially, the database explicitly links ASRM events to nearby heterozygous SNPs within isoform context, enabling systematic evaluation of variant–modification associations. In addition, DirectASRM integrates extensive functional annotations from both intra-sample analyses and external resources, providing a unified platform for exploring the regulatory and disease relevance of allele-specific epitranscriptomic variation.

## 2 Materials and methods

### 2.1 Data collection

In DirectASRM, we curated 109 direct RNA sequencing (DRS) samples from 25 independent studies across nine diploid species, collected from the Gene Expression Omnibus (GEO), the European Nucleotide Archive (ENA), and the National Genomics Data Center (NGDC) (Table S1) (Clough *et al.* 2024, Yuan *et al.* 2024, CNCB-NGDC Members and Partners *et al.* 2025). The corresponding references for each species are summarized in Table S2.

### 2.2 Detection of allele-specific RNA modifications events

Raw FAST5 data were base-called using Guppy v7.1 and aligned to reference genomes with Minimap2 (Li 2018). SNPs were identified using Longshot (Edge and Bansal 2019) and filtered to exclude variants in UCSC RepeatMasker microsatellite regions (Tarailo-Graovac and Chen 2009) or known RNA editing sites (Ramaswami and Li 2014). Germline variants were retained based on species-specific databases, while somatic variants were defined by comparison with matched controls or, when controls were unavailable, by excluding known germline variants (Table S3).

To minimize alignment bias near variant sites, reads were realigned to SNP-masked transcriptome references (Park and Cenik 2025). Nanopolish v0.13.3 was used to align raw nanopore signal data to reference transcripts (Simpson *et al.* 2017). Reads overlapping informative SNPs were assigned to reference and alternative allele–specific BAM files (Park and Cenik 2025), which were subsequently used for allele-specific m^6^A detection with m6Anet (Hendra *et al.* 2022) and general ASRM identification using the xPore diffmod module (Pratanwanich *et al.* 2021). Identified significantly ASRM sites were annotated by integrating curated RNA modification databases, including DirectRMDB (Zhang *et al.* 2023), RMBase 3.0 (Xuan *et al.* 2024), and DirectRM (Zhang *et al.* 2025), while sites without confident annotation were labeled as undetermined. Additional details are provided in the Supplementary Materials.

### 2.3 Evaluation of ASRM-SNP pair association

To evaluate ASRM–SNP associations, we examined all SNPs and ASRMs located within the same transcript. For each SNP, reads from the reference and alternative alleles were stratified by modification status based on estimated modification stoichiometry, and the numbers of modified and unmodified reads were quantified for each allele. Fisher’s exact test was then applied to assess allelic differences in RNA modification levels. In addition, independent support from external NGS-based RNA modification resources, including m6A-ATLAS v2.0 (Liang *et al.* 2024), m7GHub v2.0 (Wang *et al.* 2024), OpenAc4C (Tu *et al.* 2026), m5C-Atlas (Ma *et al.* 2022), RMBase v3.0 (Xuan *et al.* 2024), and RMVar v2.0 (Huang *et al.* 2025), was incorporated. Based on both statistical evidence and external support, each ASRM–SNP pair was classified into high-, median-, or low-confidence categories. Additional details are provided in the Supplementary Materials.

### 2.4 Functional annotation of ASRMs and associated variants

ASRM sites were annotated with genomic features based on the NCBI RefSeq GTF (Goldfarb *et al.* 2025) and evaluated for evolutionary conservation using phastCons scores (Siepel *et al.* 2005). Isoform-level expression was quantified using NanoCount (Gleeson *et al.* 2022). Because most publicly available direct RNA sequencing datasets lack biological replicates, GFOLD (Feng *et al.* 2012) was applied to identify transcripts or genes exhibiting significant allele-specific expression differences between reference and alternative alleles.

Associated variants were annotated with genomic features using the NCBI RefSeq GTF (Goldfarb *et al.* 2025) and assessed for conservation using phastCons scores (Siepel *et al.* 2005). Variant deleteriousness was evaluated by integrating prediction scores from multiple algorithms, including SIFT (Kumar *et al.* 2009), PolyPhen2 HVAR and PolyPhen2 HDIV (Adzhubei *et al.* 2010), LRT (Chun and Fay 2009), and FATHMM (Shihab *et al.* 2013), through the ANNOVAR framework (Wang *et al.* 2010). Variant functional consequences, such as synonymous and nonsynonymous changes, were also annotated using ANNOVAR (Wang *et al.* 2010). To investigate the potential involvement of RM-associated variants in post-transcriptional regulation, NanoSplicer (You *et al.* 2022) was employed to detect intra-sample allele-specific splicing events uniquely present in either the reference or alternative allele. Additional regulatory context was explored by intersecting variant loci with curated resources, including RBP binding sites from POSTAR3 (Zhao *et al.* 2022), miRNA target sites from miRanda (Agarwal *et al.* 2015) and starBase v2.0 (Li *et al.* 2014), and circular RNA annotations from CIRCpedia (Dong *et al.* 2018).

### 2.5 Disease and trait association of allele-specific RNA modification variants

To investigate potential epitranscriptome-related pathogenesis, we integrated the ClinVar (Landrum *et al.* 2020) and trait-associated tagSNPs from GWAS Catalog (Sollis *et al.* 2023). In addition, ASRM-associated somatic variants were cross-referenced with three major cancer genomics databases: TCGA (release v35) (Weinstein *et al.* 2013), COSMIC (Forbes *et al.* 2010) and ICGC (Hudson *et al.* 2010).

### 2.6 Database and web interface implementation

Metadata are stored and managed using MySQL. The web interface was implemented using HTML, CSS, and PHP, and integrates the JBrowse2 genome browser for interactive data visualization (Buels *et al.* 2016).

## 3 Results

### 3.1 Database content

DirectASRM identifies 55 399 genetic variants that may conditionally affect (add or remove) RMs (99 070 gain-ASRM and 90 953 loss-ASRM events) across 14 RNA modification types in nine species (Table S4). In total, 1 268 997 SNP-ASRM associations were detected within transcripts, including 993 323 low-, 153 509 median-, and 122 165 high-confidence pairs (Table S5), with comprehensive functional annotations summarized in Tables S6 and S7. The identification and confidence classification workflow of SNP-ASRM associations is shown in Fig. S1. Notably, both ASRM-associated variants and ASRM sites are significantly more evolutionarily conserved than their non-ASRM counterparts in humans (KS test, *P* < 1 × 10^−4^, Fig. S2), highlighting their potential functional importance.

**Figure 1:**
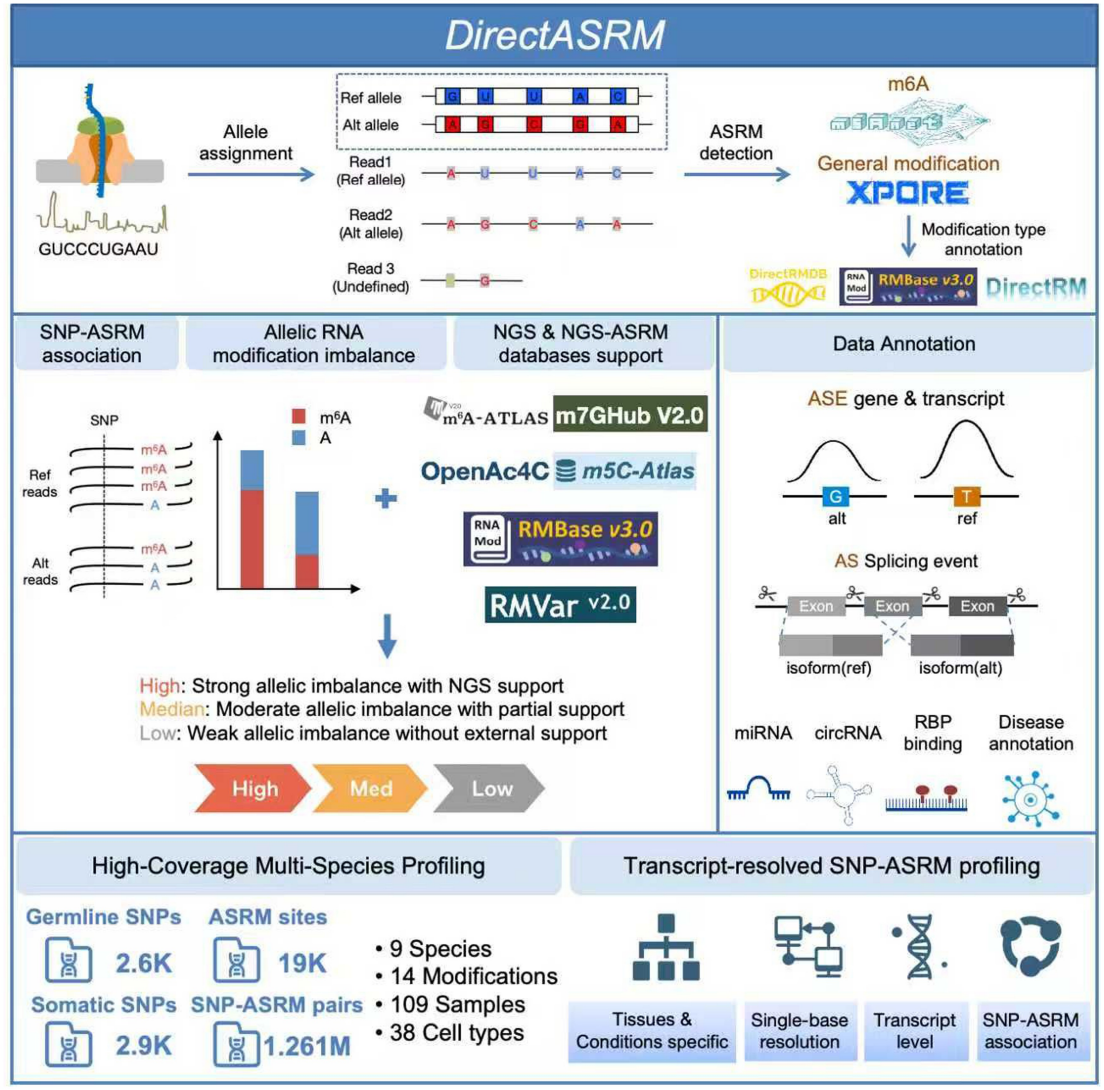
The overall design of DirectASRM. DirectASRM integrates direct RNA sequencing data to identify allele-specific RNA modification (ASRM) events at single-base and transcript resolution. ASRM–SNP associations are evaluated based on allelic modification imbalance and supported by independent NGS-based RNA modification databases, followed by comprehensive functional annotation. The database enables transcript-resolved, multi-species profiling of genetic effects on RNA modifications across diverse tissues and conditions.

An overview of DirectASRM is illustrated in Fig. 1. Compared with existing resources such as RMVar 2.0 and RMpore, DirectASRM provides broader species coverage (9 vs. 2), supports a larger spectrum of RNA modification types (14 vs. 9 and 7), and uniquely integrates transcript-level SNP–ASRM associations derived from direct RNA sequencing. In addition, DirectASRM enables intra-sample allele-specific annotation, a feature not available in RMVar 2.0 or RMpore, thereby facilitating more refined interpretation of genetic effects on RNA modifications (Table S10). A comparative Venn diagram showing the overlap between DirectASRM and existing resources is presented in Fig. S3.

### 3.2 Website interface and usage

DirectASRM provides a searchable, browsable, and downloadable web interface, integrating a genome browser for interactive exploration. All data are freely available, with comprehensive usage documentation provided.

## 4 Conclusion

DirectASRM provides a unified framework for characterizing allele-specific RNA modifications and their associated genetic variants. By integrating direct RNA sequencing–based evidence with independent NGS-based RNA modification resources and comprehensive functional annotations, DirectASRM enables systematic evaluation of genetic effects on RNA modification landscapes. This resource supports epitranscriptome-informed interpretation and prioritization of functional genetic variants and facilitates mechanistic investigations of post-transcriptional regulatory processes. Further discussion of limitations is provided in the Supplementary Materials.

## Supplementary Material

btag432_Supplementary_Data
