# Supplementary material for: DirectASRM: uncovering allele-specific post-transcriptional RNA modifications through direct RNA sequencing
Source: Bioinformatics. 2026 Jun 22;42(7):btag432. doi: 10.1093/bioinformatics/btag432 (PMC13350993; doi:10.1093/bioinformatics/btag432)
Supplement: btag432_Supplementary_Data [file btag432_supplementary_data.zip › Supplementary material.docx]

**Supplementary methods**

**2.2 Detection of allele-specific RNA modifications events**

**To minimize alignment bias near variant sites, we constructed an SNP-masked transcriptome reference in which all identified single-nucleotide polymorphism (SNP) positions were replaced with ‘N’ using genome-derived coordinates. Nanopore reads were then realigned to the SNP-masked transcriptome using minimap2 (Park and Cenik, 2025).** Raw nanopore signal data were aligned to the SNP-masked transcriptome reference using Nanopolish (v0.13.3) with the eventalign function (Simpson *et al.*, 2017). Allelic origin of reads was determined using the allele_assignment.py script developed by Park and Cenik (Park and Cenik, 2025). Briefly, SNP positions overlapped by each read were identified based on the alignment CIGAR string, explicitly accounting for insertions and deletions, while SNPs located within deletion regions were excluded. For each read, the numbers of reference and alternative alleles among covered heterozygous SNPs were counted, and only reads with at least three informative SNPs (ref_count + alt_count > 2) were retained for allele assignment. An allele bias ratio was then calculated as alternative SNP count / (reference SNP count + alternative SNP count). Reads with an allele bias ratio ≥ 0.6 were classified as alternative allele reads, whereas those with an allele bias ratio ≤ 0.4 were classified as reference allele reads. Reads with intermediate ratios (0.4 < ratio < 0.6) or insufficient SNP coverage were classified as undefined and excluded from downstream analyses to ensure high-confidence allele assignment.

For allele-specific m6A detection, m6Anet (Hendra *et al.*, 2022) was applied independently to reference and alternative allele BAM files. Only sites supported by a minimum coverage of 20 reads in both allelic groups were retained. A site was classified as a gain event if the alternative allele showed evidence of modification (probability_modified_alt > 0.5) and exhibited at least a 1.5-fold higher methylation level compared to the reference allele (modified_ratio_alt / modified_ratio_ref ≥ 1.5). Conversely, a site was classified as a loss event if the reference allele was likely modified (probability_modified_ref > 0.5) and showed at least a 1.5-fold higher methylation level than the alternative allele (modified_ratio_ref / modified_ratio_alt ≥ 1.5). In parallel, general allele-specific RNA modification (ASRM) events were detected using the diffmod module of xPore (Pratanwanich *et al.*, 2021), which directly compares modification signals between reference and alternative alleles. Multiple testing correction was performed using the Benjamini–Hochberg procedure, and sites with a false discovery rate (FDR) < 0.05 were considered significant. For each annotated ASRM site, gain or loss was assigned based on whether the alternative allele exhibited a higher or lower estimated modification rate relative to the reference allele.

**2.3 Evaluation of ASRM-SNP pair association**

**To evaluate ASRM-SNP pair association, we examined all SNPs and ASRMs located within the same transcripts. Only SNP sites supported by a minimum of 10 reads in both reference and alternative allelic groups were retained. For each SNP, reads were stratified by allelic origin and modification status based on estimated modification stoichiometry, and the numbers of modified and unmodified reads were quantified for each allele. *Fisher’s exact test* was then applied to assess allelic differences in RNA modification levels.** Multiple testing correction was performed using the Benjamini–Hochberg procedure. Statistical allelic imbalance levels were first assigned based on both multiple-testing–adjusted q-values and empirical quantiles of the absolute odds ratio (|OR|) distribution. Associations were classified as high if they satisfied q < 0.05 and ∣OR∣ ≥ Q_0.9_(∣OR∣), median if q < 0.2 and ∣OR∣ ≥ Q_0.6_(∣OR∣) and low if ∣OR∣ ≥ Q_0.6_(∣OR∣) without meeting the higher significance thresholds. These initial statistical levels were subsequently refined using independent evidence from external NGS-based RNA modification databases.

Independent NGS support was quantified using a rule-based scoring scheme (**Table S9**).

**Table S8 NGS-based support scoring for ASRM-SNP association.**

| **Evidence type** | **Criterion** | **Score** |
| --- | --- | --- |
| NGS | ASRM site within MeRIP peak and ≤50 nt from summit | +2 |
|  | ASRM site within MeRIP peak but >50 nt from summit | +1 |
|  | Single-nucleotide support within 15 nt | +2 |
|  | Replication across ≥2 independent datasets | +1 |
|  | Tissue or cell-type match (or close lineage) | +1 |
| NGS–SNP (RMVar v2) | SNP within ASRM-associated MeRIP peak and ≤50 nt from summit | +2 |
|  | Exact SNP position and allelic direction match | +1 |
|  | Replication across ≥2 independent datasets | +1 |
|  | Tissue or cell-type match (or close lineage) | +1 |

Final confidence levels were assigned by jointly considering statistical allelic imbalance and external NGS-based support (**Table S10**).

**Table S9 Integrated confidence level assignment for ASRM-SNP association.**

| **Statistical level** | **NGS / NGS–SNP support** | **Final confidence** |
| --- | --- | --- |
| High | NGS score ≥2 or NGS–SNP score ≥2 | High |
| Median | NGS score ≥3 or NGS–SNP score ≥3 | High |
| High | NGS score <2 and NGS–SNP score <2 | Median |
| Median | NGS score ≥1 or NGS–SNP score ≥1 | Median |
| Low | NGS score ≥3 or NGS–SNP score ≥3 | Median |
| Median | NGS score = 0 and NGS–SNP score = 0 | Low |
| Low | NGS score <3 and NGS–SNP score <3 | Low |

**Supplementary results**

**3.1 Database content**

**Figure S1**


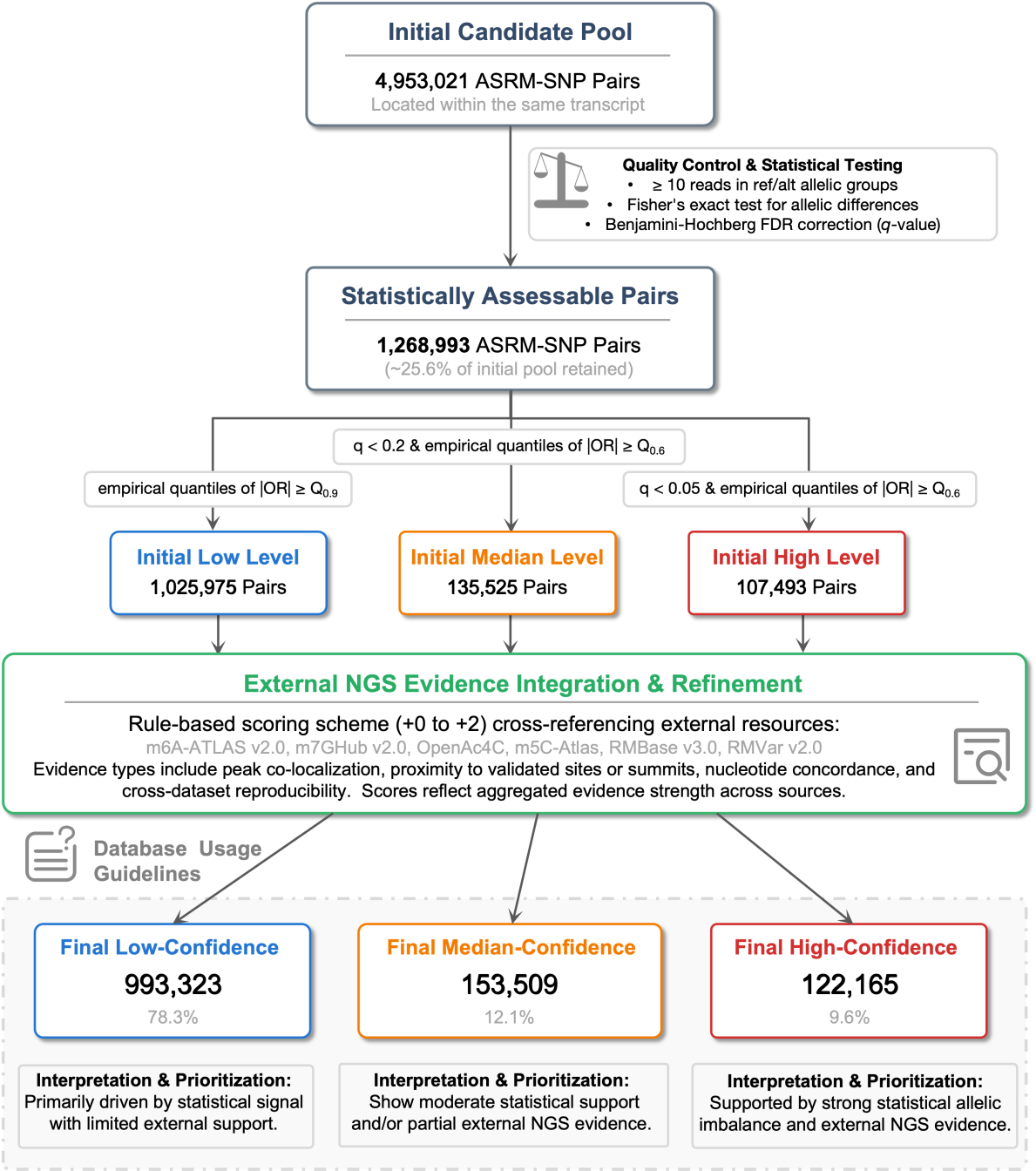


**Figure S1. Workflow for identification and confidence classification of SNP–ASRM associations. A total of 4,953,021 candidate SNP–ASRM pairs within the same transcript were identified, of which 1,268,993 (~25.6%) passed read depth filtering (≥10 reads per allele), Fisher’s exact test, and FDR correction. These associations were initially stratified based on q-value and |OR| quantiles, followed by integration of external NGS evidence (e.g., m6A-ATLAS, RMBase, RMVar) using a rule-based scoring framework. Final classifications include low-confidence (993,323; 78.3%), medium-confidence (153,509; 12.1%), and high-confidence (122,165; 9.6%), reflecting increasing levels of support from independent datasets.**

We implemented a two-stage framework to classify SNP–ASRM associations by integrating statistical evidence with external validation. All SNP–ASRM pairs within the same transcript (~4.95 million) were initially considered. After applying read coverage filters (≥10 reads per allele) and statistical testing (Fisher’s exact test with FDR correction), ~1.27 million pairs (~25.6%) were retained. These were stratified into initial tiers based on q-value and effect size (|OR|).

External NGS-based evidence (e.g., m6A-ATLAS, RMBase, RMVar) was then incorporated using a rule-based scoring framework, considering co-localization, proximity to validated sites, and cross-dataset support. Final confidence levels reflect combined evidence strength: high-confidence associations are supported by strong statistical signals and external validation, medium-confidence associations show moderate support, and low-confidence associations rely primarily on statistical signals.

We recommend prioritizing high-confidence associations for downstream analyses, while medium- and low-confidence sets can be served as candidates for further validation.

**Figure S2**

**
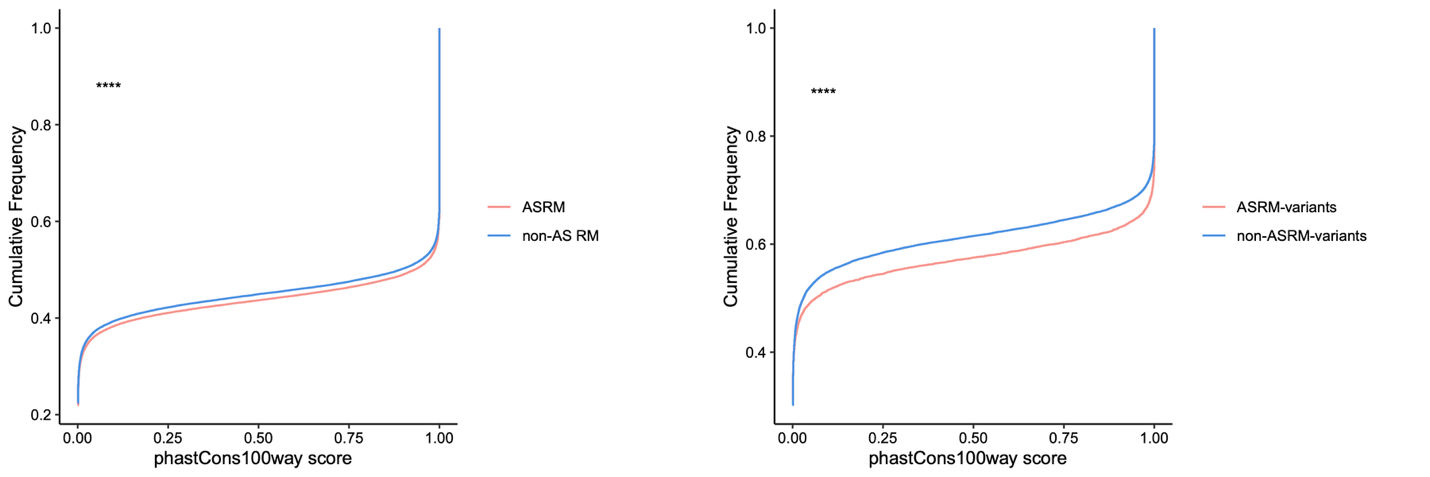
**

**Figure S2. The comparison between ASRM-related sites and variants with background counterparts. Cumulative distribution of phastCons100way conservation scores in human comparing ASRM sites with non-ASRM RNA modification sites (left) and ASRM-associated variants with non-ASRM variants (right). ASRM-related sites and variants show significantly higher evolutionary conservation than their non-ASRM counterparts (****, p < 1 × 10⁻⁴). The *P* value of the cumulative distribution curve was calculated using the *Kolmogorov–Smirnov (KS)* test.**

**Table S10 ASRM in DirectASRM compared with RMVar 2.0 and RMpore**

|  | **DirectASRM** | **RMVar 2.0** | **RMPore** |
| --- | --- | --- | --- |
| Species covered | 9 | 2 | 2 |
| RNA modification types | 14 | 9 | 7 |
| ASRM sites | 190,023 | - | 114,237  (Haplotype-biased) |
| SNP sites | 55,399 | 79,156 | - |
| SNP-ASRM association pairs | 1,261,331 | - | - |
| Sequence technology | ONT | NGS | ONT |
| Transcript-level resolution | Yes | - | Yes |
| Intra-sample annotation | Yes | - | - |

**Figure S3**

**
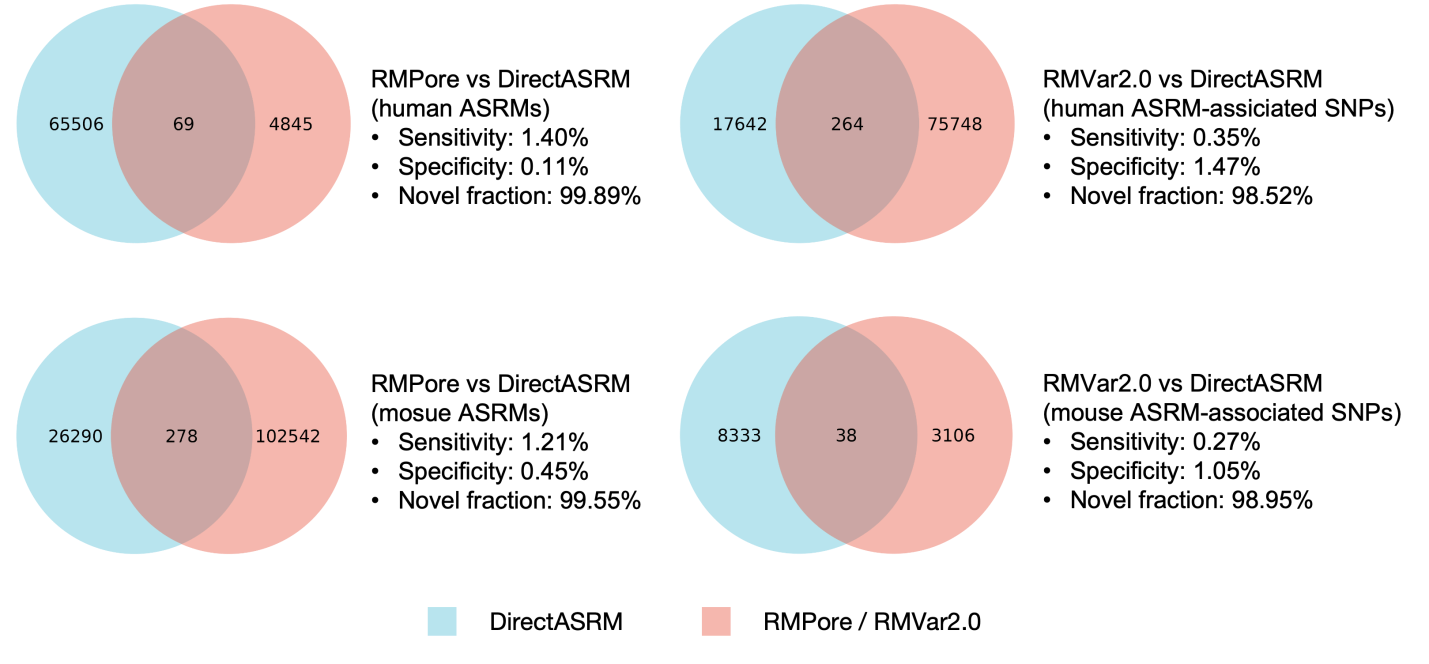
**

**Figure S3. Comparative Venn Diagram Depicting Overlap of DirectASRM with Existing Databases. DirectASRM** is illustrated in blue, while existing databases (RMPore/RMVar2.0) are demonstrated in red. Concordance is evaluated using sensitivity (overlap relative to external resources), specificity (overlap relative to DirectASRM) and novel fraction (non-overlapping proportion of DirectASRM).

**5. Limitations**

Reference mapping bias is a key concern in allele-specific analyses. In this study, we employed SNP masking strategy to mitigate reference bias in long-read RNA sequencing; however, it may lead to partial read loss and does not fully eliminate mapping bias, particularly in regions containing nearby indels or complex haplotypes. In addition, **our allele assignment strategy also does not explicitly reconstruct haplotypes and may therefore be less precise than haplotype-aware approaches in highly complex regions. Although requiring multiple informative SNPs per read improves assignment robustness, some reads remain ambiguously assigned and are excluded from downstream analyses, potentially reducing sensitivity for certain ASRM events. Furthermore, ASRM detection may be influenced by basecaller choice. We used Guppy v7.1 for consistency, while future updates may incorporate improved basecallers. Finally, as this work focuses on building a comprehensive resource, independent experimental or orthogonal computational validation will be important for downstream studies.**

**References**

Hendra,C. *et al.* (2022) Detection of m6A from direct RNA sequencing using a multiple instance learning framework. *Nat Methods*, **19**, 1590–1598.

Park,D. and Cenik,C. (2025) Long-read RNA sequencing reveals allele-specific N6-methyladenosine modifications. *Genome Res.*, **35**, 999–1011.

Pratanwanich,P.N. *et al.* (2021) Identification of differential RNA modifications from nanopore direct RNA sequencing with xPore. *Nat Biotechnol*, **39**, 1394–1402.

Simpson,J.T. *et al.* (2017) Detecting DNA cytosine methylation using nanopore sequencing. *Nat Methods*, **14**, 407–410.
